# GOMEA: A Conceptual Design of a Membrane Electrode Assembly for a Proton Exchange Membrane Electrolyzer

**DOI:** 10.3390/membranes13070614

**Published:** 2023-06-21

**Authors:** Torsten Berning, Dmitri Bessarabov

**Affiliations:** 1 Department of Energy, Aalborg University, 9220 Aalborg, Denmark; 2 Hydrogen South Africa Infrastructure (HySA), Faculty of Engineering, North-West University, Potchefstroom 2520, South Africa

**Keywords:** PEM water electrolysis, membrane–electrode assembly, graphene oxide, GOMEA, pressure difference, water and hydrogen crossover

## Abstract

We are proposing a conceptual membrane electrode assembly (MEA) of a proton exchange membrane water electrolyzer that includes a layer of graphene oxide (GO) at the cathode side. This GO layer primarily reinforces the MEA to allow operation at a higher pressure difference between the cathode and anode side. Additional benefits would be that a perfect GO layer would prevent both water and hydrogen crossover and thus would allow for pure, dry hydrogen escaping directly from the electrolyzer without losses due to hydrogen crossover, thus eliminating the need for hydrogen clean-up steps. The mechanical strength of graphene will also allow for a thinner polymer electrolyte membrane and could thus save cost. Finally, the effect of electro–osmotic drag on the water content in such an MEA is discussed, and it is argued that it could lead to an oversaturated membrane, which is highly desirable.

## 1. Introduction

Proton exchange membrane (PEM) water electrolysis is becoming of increasing interest as there is a growing demand for hydrogen as an energy carrier to store intermitted energy sources like solar and wind [1]. Compared to competing electrolyzer technologies such as alkaline electrolyzers or solid oxide water electrolysis, PEM technology has the advantage of quick start-up times and a higher hydrogen production pressure, while disadvantages are cost-related due to the need for precious metals for the catalysts and titanium bipolar plates which have the added disadvantage of difficult manufacturability [2]. The modularity of PEM electrolyzers makes them especially attractive for offshore applications in combination with wind turbines. A comparing expert opinion overview has suggested that PEM technology can become dominant, provided the cost can be reduced [3]. The expert opinion was that the maximum pressure in a PEM design would be 200 bar, compared to 30 bar and 25 bar for alkaline and solid oxide technology, respectively. As early as 2005, a commercial PEM electrolyzer stack with a delivery pressure of 200 bar by Proton Energy Systems, Inc., Wallingford, CT, USA (e.g., [4]) was available.

While the difficulty of producing hydrogen at even higher pressures seems to have been overcome by the Honda design that can produce hydrogen at 700 bar, the general cost is still high because of the need for precious metals. Two of the main technical problems with PEM electrolyzers are hydrogen crossover from the cathode side to the anode side, which becomes a significant loss mechanism when the hydrogen pressure exceeds 100 bar [4], and water crossing from the anode to the cathode and diluting the hydrogen. Even when the hydrogen pressure is in the range of a few hundred bars, which leads to less water vapor dilution, there is still a need for a hydrogen purification step. Thus, if an internal barrier to hydrogen and water crossover could be conceived, pure and dry hydrogen could be produced directly inside the electrolyzer at an increased efficiency and without needing a downstream purification step, thus saving cost.

The proton exchange membrane in these electrolyzers is typically of the Nafion type, and the general structure of a Nafion membrane is described in various textbooks on fuel cells and electrolyzer technology (e.g., [5,6]). Nafion consists of a backbone similar to polytetrafluoroethylene (Teflon) to provide mechanical strength. Unlike Teflon, it also includes sulfonic acid (SO^3−^H^+^) functional groups to ensure proton conductivity. The denotation in Nafion membranes includes the inverse number of sulfonic acid groups per unit weight (equivalent weight) and the membrane thickness in thousands of an inch. For example, Nafion 117 has an equivalent weight of 1100 g/mole and is 7 mills (0.178 mm) thick.

The proton conductivity of Nafion depends strongly on the water content of the membrane [7], and therefore Nafion has to be hydrated and is permeable to water. While it is generally believed that this water transport is a complex combination of different transport mechanisms such as electro–osmotic drag (EOD), “back” diffusion, hydraulic permeation, and possibly even thermo–osmotic drag [8], Berning suggested that in the absence of a large pressure gradient, any water that is dissolved in the membrane phase simply diffuses through the membrane [9]. Berning proposed a hybrid model where the membrane water is either dissolved water which is part of the membrane phase, or “free” water that can be pressed through the membrane. The difference between these two types of water is the measurable energy of phase change because the water that becomes part of the membrane phase is said to be condensed into the membrane. It is known that the hydraulic permeability in Nafion is a strong function of the water content [10,11].

The Nafion membrane is also permeable to gases like hydrogen and, to a lesser degree, oxygen. Especially in a PEM electrolyzer, it is important to prevent a high amount of hydrogen crossover because it is a large loss mechanism, and it raises safety concerns as the hydrogen will mix with the oxygen at the anode side and could result in an explosive mixture.

## 2. Conventional Methods of Reducing Gas Crossover in Membranes

Hydrogen and oxygen crossover phenomena through a solid polyelectrolyte membrane constitute one of the important safety concerns in PEM electrolysis. Mitigation strategies to reduce gas crossover may include various approaches based on the interaction mechanism of hydrogen and the membrane matrix. An overview of the hydrogen transport mechanisms in a PEM membrane was given by Bessarabov and Millet [12]. These authors distinguished between a passive approach and a reactive approach and a combination thereof. The passive approach reduces the crossover of the reactants via chemical permeation, which depends on the solubility and the diffusion coefficient, as well as the membrane thickness. However, a general disadvantage of reducing the reactant crossover is typically that the proton conductivity of the membrane decreases as well, leading to larger ohmic losses. In the reactive approach, hydrogen consumption is promoted via the placement of platinum catalyst either directly inside the membrane or in other parts of the cell. More details are discussed in reference [12].

While both the passive and the reactive approaches are promising when operating at a low-pressure gradient, it is generally desirable to operate a PEM electrolyzer at a high-pressure difference between the hydrogen side and the water/oxygen side. Therefore, the membrane should not just prevent hydrogen crossover but must also withstand high mechanical stress. For this application, an alternative to changing the membrane structure—which in all likelihood will add to the cost while reducing proton conductivity—might be to add a separate layer of graphene oxide that adds mechanical stability and potentially prevents hydrogen crossover. An overview of attempts to use graphene or graphene oxide in fuel cells or electrolyzers is given next.

## 3. Graphene and Graphene Oxide in Fuel Cells and Electrolyzers

**Figure 1:**
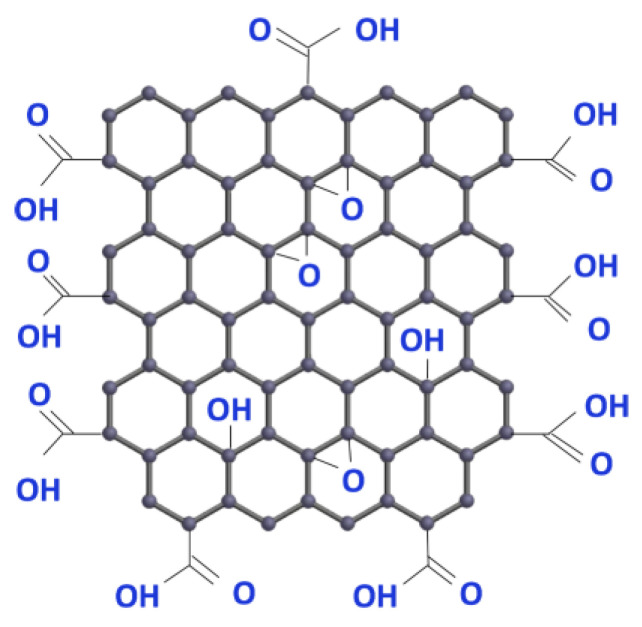
The graphene oxide molecular structure consists of carbon, hydrogen and oxygen. Image used with permission of ACS Material (https://www.acsmaterial.com).

Graphene has already been proposed by some authors as a key material for the use in fuel cells and electrolyzers. Graphene technology is advancing rapidly, and more applications can be found continually. Graphene oxide can be made from graphite and is claimed to be inexpensive. An example of its molecular structure is shown in Figure 1.

The patent “Electrochemical cell containing a graphene-coated electrode” in 2016 generally described the use of graphene in the electrodes of electrochemical cells, in that case predominantly alkaline electrolyzers [13].

While a perfect graphene layer cannot be penetrated by any materials, Geim et al. discovered that protons could penetrate a layer of oxidized graphene [14]. Consequently, the use of a layer of oxidized graphene coated to the polymer electrolyte membrane for use in direct-methanol fuel cells was proposed and investigated by Holmes et al. [15]. This group measured a performance increase of 50% and a reduced methanol crossover loss. They also found that the addition of chemical vapor deposition graphene (above 50 °C) and hexagonal boron nitride (at all temperatures) into the MEA has demonstrated no change in proton conductivity but lower fuel permeability [16].

There are only a few mentions of the use of graphene in electrolyzers. In 2013, Raman et al. [17] studied graphene-coated electrodes in an alkaline anion exchange water electrolyzer and found that the performance exceeded that of standard electrodes. In 2016, Cai et al. [18] studied the performance of a microbial electrolysis cell with a graphene-coated nickel-foam electrode at the hydrogen side and found a higher activity. In 2018, Yoshikazu et al. [19] reported the use of holey graphene for the hydrogen evolution reaction in a PEM electrolyzer in order to replace the use of precious metals. Their electrode outperforms one with non-holey graphene and is reportedly similar in performance to the standard Pt/C electrode.

The literature study on the use of graphene-based non-precious metal catalysts for the hydrogen evolution reaction was presented by Luis-Sunga et al. [20]. Thangavel et al. [21] used graphene nanoplatelets to support a NiFe electrode at the anode side of an alkaline anion exchange membrane water electrolyzer. They reported a performance of 540 mA/cm^2^ at a voltage of 1.85 V and a temperature of 70 °C.

Very recently, Garcia et al. [22] described in a book chapter the use of graphene in the catalyst layers of fuel cells and electrolyzers, including a fundamental study of the mechanism and kinetics of hydrogen and oxygen evolutions, as well as oxygen reduction and hydrogen oxidation on graphene-based catalysts in a wide pH range.

Thus, much prior work has focused on applying graphene or graphene oxide inside the fuel cell or electrolyzer catalyst layers, but only a few publications exist on the use of graphene oxide to function as a membrane replacement. In addition to the work by Holmes et al. [15], the application of graphene oxide to replace or enhance the membrane was studied by Kida et al. [23], who investigated water vapor electrolysis using graphene oxide (GO) nanosheets instead of a (Nafion) membrane. The thickness of these nanosheets was around 180 microns, which is similar to Nafion 117. The measured proton conductivity of such GO sheets was around 3.5 mS/cm at 40 °C but decreased by an order of magnitude at higher temperatures. By comparison, Nafion has a proton conductivity of around 10 mS/cm, which increases with temperature under humidified conditions [24]. Moreover, as mentioned above, the proton conductivity of Nafion exhibits a strong dependency on the membrane water content [7].

In a very recent work, Ravikumar et al. [25] described an electrolyzer that operates on atmospheric, humid air without the need to supply water due to the extraordinary water sorption kinetics of GO. They obtained a current density of around 165 mA/cm^2^ at a terminal voltage of 2 V at atmospheric conditions. 

Additionally, Diaz-Abad et al. [26] investigated the addition of graphene oxide to polybenzimidazole (PBI) membranes as an organic filler with different weight contents for use in water electrolysis at higher temperatures. The addition of GO resulted in better proton conductivity of the membrane, better phosphoric acid retention capabilities, and improved water management. It was also observed that three times more hydrogen is produced when the GO weight content is 2% compared to a non-modified membrane.

Finally, Bal et al. [27] have studied the electrochemical hydrogen purification with a graphene-supported RuPt catalyst and obtained promising results.

In summary, while there has been increasing focus on using graphene or graphene oxide in the electrodes of fuel cells or electrolyzers, only a few studies have focused on employing graphene oxide in addition to or instead of the proton exchange membrane. However, the opportunities this would offer are amazing, as will be described below. 

## 4. The Use of Oxidized Graphene in PEM Electrolyzers: Opportunities and Challenges

While the opportunities for the use of graphene in low-temperature fuel cells are high, and clear improvements in terms of performance have already been demonstrated by Holmes et al. [15], the opportunities in electrolyzers appear to be even larger because here, the addition of oxidized graphene to the membrane can lead to a breakthrough in this technology. 

**Figure 2:**
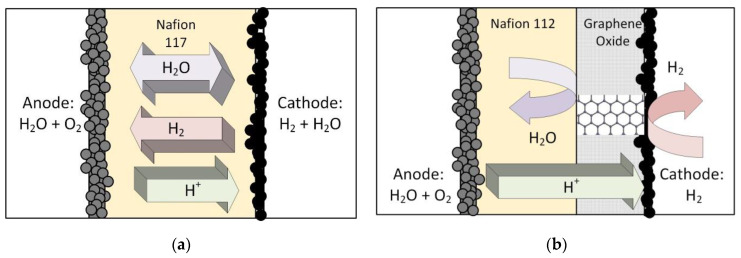
(**a**) Standard PEM electrolyzer MEA that allows for water transport, hydrogen crossover, and proton migration. (**b**) Modified MEA with a graphene oxide layer (GOMEA). Ideally, this only allows proton migration and permits a thinner, cheaper Nafion membrane.

Figure 2 shows a standard, single catalyst layer PEM electrolyzer MEA and a conceptual design of an MEA with a layer of oxidized graphene at the cathode side. It is generally accepted that carbon-based material cannot be used at the anode side because of corrosion (e.g., [28,29]), but at the cathode side, the use of graphene should be unproblematic. The underlying idea in this work was to find a way to mechanically reinforce the electrolyzer MEA to make it suitable for a high-pressure gradient across the membrane. For this purpose, reinforced polymer electrolyte membranes such as Nafion 324 have been developed, but the cost of these membranes is substantially higher compared to the standard Nafion 117, which is frequently used. Compared to the thickness of 178 mirons of Nafion 117, Nafion 324 has a thickness of 280 microns and, due to the higher Teflon content, has a higher mechanical strength and can thus withstand a higher pressure gradient. Clearly, this comes at the cost of lower proton conductivity in addition to the higher price. Therefore, it was deemed desirable to find an alternative to a reinforced membrane.

Reinforcing the 10-micron-thick catalyst layer(s) instead of the membrane has only become an alternative due to the recent advancements in graphene technology, and there are very clear advantages, in addition to avoiding a reinforced membrane and the associated disadvantages:

1. A perfect, oxidized graphene layer will only allow for the penetration of protons. Holmes et al. [16] have found that even at elevated temperatures, there is no reduction in proton conductivity when employing a GO layer. Thus, hydrogen crossover can be reduced and ideally prevented altogether. Especially at a cathode pressure above 100 bar, hydrogen crossover becomes a large loss mechanism, and this is the reason why some electrolyzer manufacturers are aiming to operate their devices at a current density of 10 A/cm^2^ [30] in order to keep the faradaic losses small compared to the current drawn. Such high current density operation must, however, come at the cost of lower efficiency;
2. By the same argument, a perfect layer of oxidized graphene will also prevent water from crossing over from the anode to the cathode and diluting the hydrogen. If water crossover could be prevented, pure hydrogen could be created in the electrolyzer, and there would be no necessity for a downstream clean-up step, thus saving cost and reducing the system’s complexity. While this advantage is not on par with the above-mentioned one, it is still noteworthy;
3. If the graphene layer provides the needed mechanical strength, a thinner membrane could be used instead of the typical Nafion 117. The cost of the membrane is directly related to its thickness, and of course, a thinner membrane will also lead to a lower protonic loss inside the membrane.

While these advantages are compelling, one must not forget that Nafion membranes show swelling of up to 15% between the dry and the hydrated state [24], and this could lead to delamination of the different layers. A further challenge can be that in their ground-breaking work, Hu et al. [14] have discovered that the proton conductivity of single-layer graphene oxide increases significantly with temperature but that no proton transport is detected for few-layer crystals. However, Holmes et al. have found a high proton conductivity in their work [16]. The interested reader is also referred to these authors for detailed insight into how a GOMEA may be manufactured.

Concerning the cost of a GO-enhanced MEA, in the literature, the term graphene oxide is typically connected to a low-cost device (e.g., [31]). The Norwegian company Abalonyx expects the cost of graphene oxide to drop by at least one order of magnitude with a wider-spread adoption of GO in various technologies and estimates a cost target of as low as EUR 22/kg at high production volumes, while currently, in battery technologies the price is around EUR 300/kg [32].

A general remark on the cost considerations is that according to the expert opinion study, the cost of a PEM electrolyzer is already slightly higher than the capital cost for competing technologies [3]. However, this study did not include any cost estimates for producing compressed hydrogen, but hydrogen produced below the storage or pipeline pressure is only of limited value. Overall, it is beyond the scope of this work to include a detailed cost analysis of an electrolyzer MEA with a GO layer because it is not yet clear how (or if) it can work.

## 5. The Role of Electro–Osmotic Drag

Upon reading that a perfect layer of oxidized graphene should also block all water transport, a first reaction might be that EOD, which is often thought of as one of the water transport mechanisms inside the membrane, could not take place, which could be a problem to electrolyzer operation. What would happen if no water could be “dragged along with the protons” through the membrane? 

It was previously proposed that EOD is, in fact, not a passive transport mechanism of water through the membrane driven by chemical-potential differences, but instead, it is a surface mechanism that drags water molecules inside and/or outside of the membrane along with the hydronium ions. It was argued that EOD could be connected to the electro-chemical reactions steps [33], as proposed, e.g., by Damjanovic et al. [34]. Should the oxidized graphene layer successfully block all water transport through the membrane, then the question would remain, at which of the electrodes EOD occurs, or even at both. This leads to different possible scenarios:

1. If EOD is only connected to the anode side reaction inside the PEM electrolyzer, e.g., the breaking of the water molecule to form oxygen, protons, and electrons, this would be the ideal scenario. It was previously pointed out that, in the case of zero net water transport through the membrane, as would be the case with an ideal graphene oxide layer, the water added to the electrolyte phase as a result of EOD must be balanced by non-equilibrium sorption (NES). The result would be a membrane that is supersaturated with water because the same amount of water that is entering the electrolyte phase must also be leaving the electrolyte phase via NES [9]. Electrolyzers are typically operating on liquid water, meaning that the equilibrium state of the membrane is highly saturated;
2. If EOD is, however, connected to the hydrogen evolution reaction, then this would be problematic for the functionality of a GOMEA because only protons would be permitted through the graphene oxide layer, and the hydrogen evolution reaction would have to function without EOD. Ye and Wang [35] have measured an EOD coefficient close to unity in a Gore-Select membrane using the hydrogen pumping technique, where the reaction mechanisms are the dissolution of hydrogen into protons and electrons at the anode side, and protons and electrons are recombined to form hydrogen at the cathode side.

Overall, there is still much work needed to fully understand the effect of EOD in the presence of the GO layer on the water electrolysis cell. What is known so far is that the previous interpretation that EOD is a water transport mechanism occurring during the polarization of the electrolysis cell inside the membrane that is balanced by other independent water transport mechanisms needs further clarification.

## 6. Conclusions

The application of graphene and graphene oxide in both fuel cells and electrolyzers offers tremendous opportunities. While most of the previous studies have focused on the electrodes, the addition of a complete layer of graphene oxide to a membrane electrode assembly (GOMEA) of a proton exchange membrane electrolyzer provides unique opportunities because, in the ideal case, dry hydrogen would be emerging from the electrolyzer and there would be no hydrogen crossover, which is a predominant loss mechanism at high-pressure operation. The added mechanical strength of graphene oxide would also allow for using a thinner Nafion-type membrane and thus save cost. The smooth Nafion membrane might provide an ideal substrate to apply the GO layer. While GO is currently still expensive, the cost can decrease by over an order of magnitude if the demand increases. Finally, a detailed cost analysis of the different types of electrolyzers should also include the cost of delivering compressed hydrogen.

## Data Availability

Not applicable.
